# High Dimensional Analyses of Circulating Immune Cells in Psoriatic Arthritis Detects Elevated Phosphorylated STAT3

**DOI:** 10.3389/fimmu.2021.758418

**Published:** 2022-01-11

**Authors:** Claudia Macaubas, Shamma S. Rahman, Idit Lavi, Amir Haddad, Muna Elias, Deepanwita Sengupta, Devy Zisman, Elizabeth D. Mellins

**Affiliations:** ^1^ Pediatrics, Program in Immunology, Stanford University, Stanford, CA, United States; ^2^ Community Medicine and Epidemiology, Carmel Medical Center, Haifa, Israel; ^3^ Rheumatology Unit, Carmel Medical Center, Haifa, Israel; ^4^ Biology, Stanford University, Stanford, CA, United States; ^5^ The Ruth and Bruce Rappaport Faculty of Medicine, Technion, Haifa, Israel

**Keywords:** psoriatic arthritis, STAT3, CD4+ T cells, monocytes, rheumatoid arthritis

## Abstract

Psoriatic arthritis (PsA) is a chronic inflammatory arthritis, affecting up to 40% of patients with psoriasis. Constitutive expression by CD4+ T cells of an active form of STAT3, a signal transducer and transcription factor, has been shown to induce many of the major features of PsA in an animal model. We used high dimensional mass cytometry (CyTOF) to probe *ex-vivo* levels of phosphorylated STAT3 (pSTAT3) in circulating immune cell subpopulations from PsA patients during active and inactive states. We evaluated the frequency of 16 immune cell populations and the levels of the activated forms of STAT3 (pSTAT3) and, for comparison, STAT1 (pSTAT1) and Src (pSrc) in whole blood fixed shortly after collection. In addition to PsA patients, we studied active rheumatoid arthritis (RA) patients. Increased levels of pSTAT3 were found in all the CD4+ T cell subsets analyzed, specifically, Th1, Th2, Th17, T follicular helper (Tfh) and T regulatory (Treg) as well as in CD14+CD16- (classical) monocytes from active PsA patients compared to inactive patients. After correcting for body mass index (BMI), smoking and conventional disease modifying antirheumatic drugs (c-DMARDs), levels of pSTAT3 levels remained increased in Th1 and Tfh CD4+ T cells, and in CD14+CD16- monocytes from active patients compared to inactive patients. No differences between the patient groups were observed for pSTAT1 or pSrc. No differences were found between the active PsA and active RA groups after correction for multiple testing. During active PsA, circulating Th1 and Tfh CD4+ T cells, and CD14+CD16- monocytes expressing high levels of pSTAT3 may play a role in PsA pathophysiology, perhaps by migration to inflamed sites.

## Introduction

Psoriatic arthritis (PsA), a chronic inflammatory arthritis belonging to the group of spondyloarthropathies, affects 6-41% of patients with psoriasis (1). A recent meta-analysis found a pooled prevalence of 133 for every 100,000 persons and an incidence of 83 for every 100,000 individuals (2). In most adult cases, arthritis develops around 10 years after the appearance of psoriasis, with the disease affecting males and females equally (3). Psoriatic arthritis also occurs in children, with distinguishing features, such as a bimodal age of onset distribution (4). PsA is a systemic disease, involving the musculoskeletal system, the skin and nails (3, 5). The term psoriatic disease has been used to encompass psoriasis, PsA and associated comorbidities (6). Psoriatic disease is associated with uveitis and inflammatory bowel disease, and an increased risk of comorbidities, especially metabolic syndrome and cardiovascular disease (7, 8). There are no specific biomarkers for PsA, and the diagnosis is based on clinical and imaging findings.

Genetic, environmental and immune-related mechanisms have been implicated in PsA pathogenesis. It is not fully understood how interactions among these different factors lead to manifestations of PsA, and initiation of the disease either at the skin site or at the entheses has been proposed (3). Many immune components, both innate and adaptive, have been associated with the inflammatory response in PsA (9). Among these, T cells and other cell types producing IL-17 have been shown to be of fundamental importance, based on evidence from genetic and tissue studies, animal models and, more recently, response to therapy (10–12). The transcription factor STAT3 has been shown to be essential for the development of Th17 cells (13). Recently, work by Yang et al. described an animal model in which overexpression of STAT3C, a constitutively active form of STAT3, in CD4+ T cells led to expression of many of the major features of PsA, including psoriasis-like skin lesions, tendinitis/enthesitis and arthritis (14, 15). This work points to a crucial role of STAT3 in PsA pathogenesis, building upon an extensive literature showing the importance of STAT3 in psoriasis (16) and PsA, including a STAT3 polymorphism (STAT3 rs744166∗G allele) that has been found to be associated with PsA (17). In a commentary to Yang et al. (14), Mountz (15) proposes a model in which dysregulation of STAT3 expression in CD4+ T cells is the ‘initiating event,’ driving both skin disease and musculoskeletal disease through the induction of Th17 and Th22 cells. In this model, Th17 cells would be mostly involved in the development of psoriatic arthritis, and Th22 cells, which express the chemokine receptor CCR10, would drive the development of psoriasis. The higher expression of STAT3 would underlie both these outcomes.

Given the complexity of the immune landscape in PsA, it is likely that, at different stages of the disease, distinct immune phenotypes can be detected and shed light on the disease immunopathology. In this study, we used the high dimensionality of mass cytometry to measure expression of phosphorylated STAT3 (pSTAT3) in non-manipulated, *ex vivo*, circulating immune populations during active and inactive PsA. We also analyzed samples from active rheumatoid arthritis (RA) for comparison. Mass cytometry, also known as cytometry by time-of-flight (CyTOF), is a novel platform combining flow cytometry and mass spectrometry that allows assessment of overall heterogeneity and degree of similarity between subsets of immune cells, based on a large number of parameters. Studies using CyTOF in rheumatic diseases have been reported (18, 19), and use of this approach in these diseases has been reviewed recently (20).

## Patients, Materials and Methods

### Patients

Demographic, clinical and laboratory parameters and treatment data were collected from 27 patients with PsA fulfilling CASPAR criteria (21) and 14 patients with RA fulfilling 2010 EULAR/ACR classification criteria (**Table 1**) (22). All patients provided informed consent, and the study was approved by the IRB at Carmel Medical Center (CMC 0044-11). The PsA study population consisted of 12 patients with inactive disease, as defined by minimal disease activity (MDA) ≥ 5, and 15 patients with active disease (MDA<5) (23). All RA patients were classified as having moderate to high disease activity, according to the clinical disease activity index (CDAI) score (24).

**Table 1:** Characteristics of the study patient population.

		Active PsA	Inactive PsA	Active RA	p-valueActive PsA - Inactive PsA^1^	p-valueActive PsA – Active RA^1^
**Number**		15	12	14		
**Age (years)^2^**		54.60±15.52	58.67±11.32	58.71±13.76	0.68	0.6
**Disease Duration Arthritis (years)^2^**		7.87±4.96	13.75±12.11	9.36±11.83	0.21	0.48
**Disease Duration: Psoriasis (years)^2^**		17.93±12.60	25.75±12.62			
**Body Mass Index^2^**		30.57±4.61	24.62± 3.36	30.29±7.18	**0.001**	0.88
**Gender(female) **		11 (73.3%)	8 (66.7%)	11 (78.6%)	1.0	1.0
**Smoking**		9 (64.3%)	2 (16.7%)	4 (28.6%)	**0.02**	0.12
**Ethnicity: Jewish**		12 (80 %)	12 (100%)	11 (78.6%)	0.23	1.0
** Arab**		3 (20.0%)	0	3 (21.4%)	0.23	1.0
**Comorbidities**						
** Hypertension**		4 (26.7%)	2 (16.7%)	5 (35.7%)	0.67	0.70
** Hyperlipidemia**		6 (40%)	4 (33.3%)	7 (50.0%)	1.00	0.59
** Diabetes Mellitus**		3 (20%)	0	0	0.23	0.22
** Asthma**		1 (6.7%)	0	1 (7.1 %)	1.0	1.0
** Ischemic Heart Disease**		2 (13.3%)	0	0		1.0
** Thyroid Diseases**		1 (6.7%)	1 (8.3%)	4 (28.6%)	1.0	0.17
**Medication**						
**c-DMARDS^3^**		5 (33.3%)	9 (75.0%)	4 (28.6%)	**0.03**	0.68
** Methotrexate**		5 (33.3%)	8 (66.7%)	4 (28.6%)	1.0	0.68
** Leflunomide**		1 (6.7%)	0	0	1.0	1.0
** Salazopyrine**		2 (13.3%)	3 (25.0%)	0	0.63	0.48
** Cyclosporine**		1 (6.7%)	0	0	1.0	1.0
** Apremilast**		1 (6.7%)	0	0	1.0	1.0
**b-DMARDS^4^**		5 (33.3%)	5 (41.7%)	0	0.71	**0.04**
**Anti TNFα**		3 (20%)	|5 (41.7%)	0	0.40	0.22
** Etanercept**		3 (20%)	3 (25.0%)	0	1.0	0.22
** Golimumab**		0	1 (8.3%)	0	0.44	1.0
** Adalimumab**		1 (6.7%)	0	0	1.0	1.0
** Infliximab**		1 (6.7%)	1(8.3%)	0	1.0	1.0
**Secukinumab**		2 (13.3%)	0	0	0.49	0.48
**Ustekinumab**		1 (6.7%)	0	0	1.0	1.0

^1^: t test or Mann Whitney test were used to compare continuous variables between two independent groups. Chi-squared test or exact test for small sample were used to compare categorical variables between groups; the bold values denote statistical significance. ^2^: Continuous variables are presented as mean ± SD. ^3^: c-DMARDS: conventional disease-modifying antirheumatic drugs; ^4^ : b-DMARDS: biological disease-modifying antirheumatic drugs. For medication, the number of patients refers to the total number of patients on the particular medication in each group.

### Samples

Four ml of blood was collected from each subject in EDTA tubes and promptly treated with Smart Tube fixative in a proportion of 1.4 parts Proteomic Stabilizer PROT1 (Smart Tube, Inc. San Carlos, CA) to 1 part whole blood, and frozen at -80°C. The fixed whole blood was thawed in a 4°C cold room. Following complete thawing, samples were diluted 1:5 with 1X Smart Tube Thaw-Lyse Buffer (Smart Tube, Inc.), mixed 5 times and incubated for 10 min at room temperature (RT). Samples were then centrifuged at 600 x g for 8 min at RT. Supernatant was decanted, and the pellet was re-suspended in 25-50 ml of Thaw-Lyse Buffer. This procedure was performed in total 3 times, followed by 2 washes with 25 ml of thaw Lyse Buffer 2 (Smart Tube, Inc). The resulting pellet was re-suspended in 1.6 ml of 0.22 μm filtered CyFacs buffer [1 x PBS (Rockland, Limerick, PA), 1% bovine serum albumin (Sigma) and 0.05% sodium azide (Sigma) in Milli-Q water] and stored at 4°C overnight in a 96 well deep well polypropylene plate (VWR, Radnor, PA).

### Antibodies

The antibody panel is shown on **Supplementary Table 1**. Antibodies were titrated using fixed blood from a healthy adult control. Antibodies were freshly combined for each experiment in a volume of 50 μl per sample and filtered using a 0.1μm Durapore PVDF filter (Millipore Sigma, St. Louis, MO) at 14,000 x g for 5 min at RT.

### Staining

The staining was performed in a 96 well deep well plate at RT. The cells, initially resuspended in 1.6 ml of CyFacs buffer, were pelleted by centrifugation at 974 x g at 4°C. Supernatant was aspirated using a manifold to control the amount of supernatant left. Following aspiration, the plate was vortexed to ensure resuspension of the cell pellet. Fc receptors were blocked using 5 μl of Human TruStain FcX™ (BioLegend, San Diego, CA) for 10 min. At the end of the incubation, 50 μl of filtered antibodies for surface antigens (**Supplementary Table 1**) was added to each well and mixed gently with the cells. The plate was incubated for 30 min at RT with gentle vortexing after 15 min. Cells were then washed twice with 1.6 ml of CyFacs Buffer and fixed with 1.6% of formaldehyde (Thermo Scientific, Rockford, IL) for 10 min at RT, followed by 2 washes with CyPBS. Cells were then permeabilized with 90% methanol (Sigma), on ice for 30 min, followed by 2 washes with CyPBS. Antibodies to intracellular antigens (**Supplementary Table 1**) were added to the cells and incubated for 30 min at RT with a gentle vortexing after 15 min, and cells were washed once with 1.6 ml of CyPBS. To enable cell identification based on DNA content, cells were labeled with 0.125 nM iridium (191Ir and 193Ir) (Cell-ID™ Intercalator-Ir, Fluidigm, South San Francisco, CA), for 20 min at RT, in a volume of 300 μl. Cells were then washed 5 times before CyTOF acquisition: 2 times with CyFacs buffer and 3 times with 0.22 μm filtered MilliQ water; the last 2 washes were performed just before acquisition.

### Mass Cytometry

A Helios mass cytometer (Fluidigm) was used for sample acquisition. Before acquisition, the machine was tuned with Tuning Solution (Fluidigm) and a bead sensitivity test was performed using EQ™ Four Element Calibration Beads (Fluidigm). The samples were resuspended in a solution of 1:10 Calibration Beads in CyWater to obtain a concentration of approximately 1 x 10^6^ cell/ml. The fluidics system SuperSampler (VictorianAirships, Alamo, CA) was used for injection of cells into the instrument. Sample files were normalized using calibration beads.

### Mass Cytometry Data Analysis

Cell subpopulations were manually determined using FlowJo version 10.5.2 (FlowJo, LLC). **Supplementary Figure 1** shows the gating strategy. Briefly, cell events (intact cells) were identified as Ir191/193 double positive events, and doublets were excluded on the basis of higher DNA content (Ir191) and longer event length. Immune cell frequencies are expressed as frequency of mononuclear cells, defined as CD45+, CD66a- cells, except for granulocytes that are expressed as frequency of total leukocytes. CD4+ T cell subsets were defined based on Kunicki et al. (25): from gated CD3+CD4+ cells, the Tfh subset was defined as CXCR5+ cells; the CXCR5- cells were then defined as follows: the Th1 subset was defined as CXCR3+ CCR4- cells, the Th2 subset as CCR4+CXCR3-, Th17 as CCR6+CD161+, and the Treg subset as CD127^lo/-^CD25^hi^ FOXP3^+^ (**Supplementary Figure 1**). For levels of phosphorylated signaling proteins, the signal intensity from a whole given population was measured, as the distribution was unimodal; the geometric mean (gmean, as defined by FlowJo software) of the signal intensity for each value was expressed as the hyperbolic arcsine of the gmean divided by a cofactor parameter (value=5) (arcsinh transformed).

### Statistical Analysis

We analyzed the differences between PsA patients at active and inactive states and the differences between the active PsA and active RA patients. Sixty-four conditions were analyzed: 16 cell subpopulations and 3 phosphorylated signaling proteins. Group to group analyses were performed using unpaired t test with Welch’s correction, using GraphPad Prism version 9.1.0.

Data were analyzed using significance analysis of microarray (SAM) analysis (26), a non-parametric method that performs correction for multiple testing, using the “samr” package in R through GitHub (https://github.com/MikeJSeo/SAM). Two class unpaired SAM analyses were used to analyze the groups, with a false discovery rate (FDR) set at <1% (q<0.01).

To analyze the contribution of variables that differed between active and inactive PsA patients, we performed linear regression model with bootstrapping for deriving robust estimates of standard errors and confidence intervals for estimates regression coefficients. This allowed estimation of the contribution of BMI, smoking and conventional disease-modifying anti-rheumatic drugs (c-DMARDS). All tests were two-sided with a p<=0.05 considered statistically significant. Statistical analyses were performed using SPSS version 24.0 (IBM, Armonk, New York, USA).

Correlations between phosphoproteins levels and disease parameters were performed using Spearman’s rank correlation in the GraphPad Prism version 9.1.0.

### Heatmaps

Heat maps were created using the Morpheus visualization and analysis software: https://software.broadinstitute.org/morpheus/. Values were transformed by subtracting row median and dividing by row median absolute deviation. Clustering was performed by hierarchical clustering using Euclidean distance with average linkage.

## Results

### Study Population

The PsA population had an average age of 58.25 ± 12.22 years, and 70% (n=19) were female. The patients with active PsA were more obese (BMI=30.57 ± 4.61) and more often smokers (64%), compared to patients with inactive PsA. Fewer active compared to inactive PsA patients were using conventional (c-)DMARDS (33% to 75%). There were no other statistically significant differences in demographic parameters, comorbidities or medications (**Table 1**) between patients with active versus inactive disease. In the active PsA group, the mean tender joint count was 14.13 ± 9.32, swollen joints 11.27 ± 6.81, Psoriasis Area and Severity Index (PASI) score 2.77 ± 2.82 and enthesitis score of 5.14 ± 7.54. The calculated minimal disease activity (MDA) score in active PsA patients was 0 in 4 patients, 1 in 8 patients and 2 in 3 patients. In the inactive PsA group, 5 patients had an MDA score of 5, 4 patients a score of 6 and 3 patients scored 7. In the RA group, 11 (79%) patients were female, and the mean age was 58.71 ± 13.76 years. The calculated mean CDAI score was 33.19 ± 14.61. No RA patient was on biologic (b)-DMARDS, compared to 33% in the active PsA group. There were no statistically significant differences in demographic and comorbidities between active PsA patients and active RA patients (**Table 1**).

### Comparisons Between Active and Inactive Psoriatic Arthritis Patients

**Figure 1:**
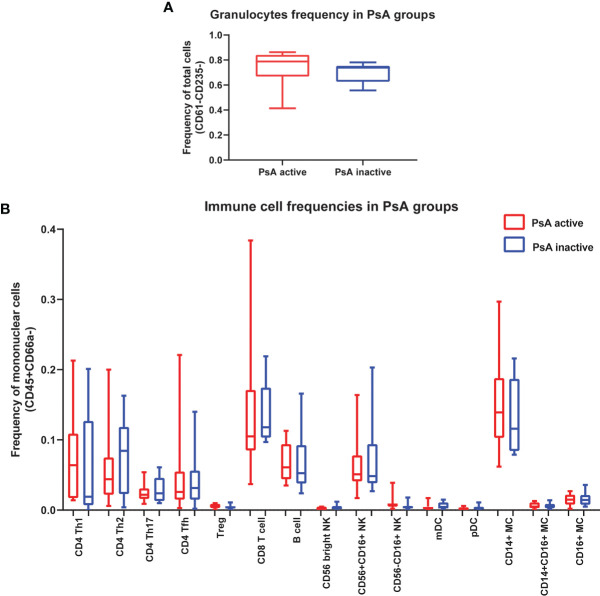
No differences in immune cell frequencies in whole blood between patients with active or inactive PsA. **(A)** Frequency of granulocytes is expressed as frequency of leukocytes. **(B)** Frequencies of 15 immune cell subpopulations expressed as frequency of total mononuclear cells (CD45+CD66a-). Data are shown as box plots extending from the 25th to 75th percentiles, and the whiskers from the minimum to the maximum point; middle line represents the median. Samples from 15 active PsA patients and 12 inactive PsA patients were tested. Group to group comparison using unpaired t test with Welch’s correction, p>0.05 in all comparisons. Tfh, T follicular helper; mDC, myeloid Dendritic cell; pDC, plasmacytoid Dendritic cell; MC, Monocyte.

**Figure 2:**
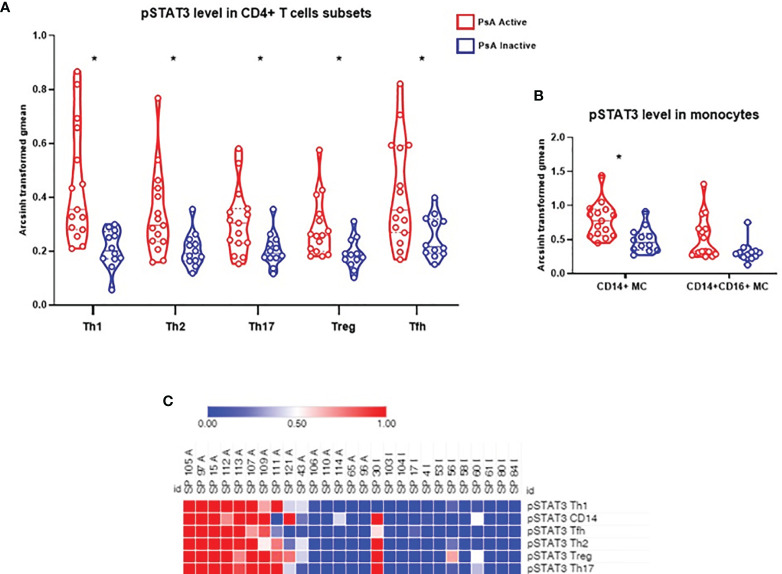
Levels of pSTAT3 are increased in CD4+ T cell subsets and in classical monocytes during active PsA. **(A)** Signal intensity of pSTAT3 in CD4+ T cell subsets from PsA patients. **(B)** Signal intensity in monocytes: CD14+CD16- (classical) subset and CD14+CD16+ (Intermediate) subset. Data expressed as arcsinh transformed geometric mean (gmean), displayed as violin plots, with the straight line representing the median and dashed lines the quartiles. Samples from 15 active PsA patients and 12 inactive PsA patients were tested. Group to group comparison using unpaired t test with Welch’s correction, all values p<0.05, see **Supplementary Tables 2**, **3** for details. *: significant difference between the 2 groups by two class unpaired SAM analysis, FDR <1% (q<0.01). **(C)** Heat map showing STAT3 phosphorylation signal in CD4+ T cell subsets and CD14+ (classical) monocytes). A, Active PsA; I, Inactive PsA; Tfh, T follicular helper; MC, Monocytes.

We compared the frequency of 16 immune cell subpopulations between active and inactive PsA. No significant differences in the frequency of these subpopulations were found between the two PsA groups, using group to group comparisons (**Figures 1A, B**). Next, we compared the level of phosphorylated proteins (pSTAT1, pSTAT3 and pSrc) in these 16 subpopulations between the two groups of PsA patients. Higher levels of pSTAT3 were found in all CD4+ T cell subsets analyzed, defined as detailed in Kunicki et al. (25), as well as in CD14+CD16- (classical) and CD14+CD16+ (intermediate) monocytes from active compared to inactive PsA patients, using group to group comparisons (**Figures 2A, B** and **Supplementary Tables 2**, **3**).

We next analyzed all data using SAM (significance analysis of microarrays): frequency of 16 immune cell subpopulations, and the baseline levels of the 3 phosphoproteins in all subpopulations, for a total of 64 comparisons. At FDR<1% (q<0.01), levels of pSTAT3 in all CD4+ T cell subsets analyzed (Th1, Th2, Th17, Treg, Tfh), as well as in CD14+CD16- monocytes (classical) were significantly different between active and inactive PsA, whereas levels in CD14+CD16+ monocytes were similar between the 2 groups (**Figures 2A, B**). Next, using the SAM results, we generated a heat map to visualize the baseline status of the 3 phosphorylated signaling proteins (pSTAT1, pSTAT3 and pSrc) in all subpopulations analyzed. As can be seen in the heat map in **Figure 2C**, the level of signaling from pSTAT3 in the CD4+ T cell subsets as well as in CD14+ monocytes are noticeably higher, albeit heterogeneous, in the active PsA group and decreased in the inactive group. In contrast, levels of pSTAT1 or pSrc did not differ between the PsA groups in the immune subpopulations assessed (**Supplementary Figure 2**).

As active PsA patients have higher level of BMI and smoking and lower level of c-DMARD usage than inactive patients (**Table 1**), we performed a bootstrap analysis that showed that levels of pSTAT3 in Th1 and Tfh CD4+ T cells, as well as in CD14+CD16- monocytes, stay significantly higher in active PsA patients, after correction for these confounding variables (**Tables 2**–**4**). The difference between the patient groups is no longer significant for Th17, Treg and Th2 CD4+ subsets after correction (**Supplementary Tables 4**–**6**).

**Table 2:** Bootstrapping analysis for pSTAT3 in CD4+ Th1 cells.

Bootstrap for Coefficients							
Model		B	Bootstrap (based on 999 bootstrap samples)				
			Bias	Standard Error	Significance (2-tailed)	95% Confidence Interval	
						Lower	Upper
1	(Constant)	3.272	.060	.594	.003	2.229	4.521
	Group	-0.940	-.026	.306	.011	-1.585	-.331
	Smoke	0.160	-.012	.413	.671	-.634	1.024
	cDMARDS	-0.713	.018	.347	.070	-1.427	-.026
	BMI	0.211	-.018	.292	.425	-.439	.780

cDMARDs, conventional disease-modifying antirheumatic drugs. For smoke and cDMARDS, the variables are yes/no for each condition. BMI, Body Mass Index; for this variable grouping was based on normal weight (below 25) versus obese (above 25).

**Table 3:** Bootstrapping analysis for pSTAT3 in CD4+ Tfh cells.

Bootstrap for Coefficients							
Model		B	Bootstrap (based on 998 bootstrap samples)				
			Bias	Std. Error	Sig. (2-tailed)	95% Confidence Interval	
						Lower	Upper
1	(Constant)	2.982	.042	.596	.001	1.862	4.338
	Group	-0.741	-.013	.346	.044	-1.464	-.041
	Smoke	-0.210	-.031	.364	.538	-.963	.503
	cDMARDS	-0.499	-.009	.362	.180	-1.306	.160
	BMI	0.299	-.002	.300	.291	-.331	.868

cDMARDs, conventional disease-modifying antirheumatic drugs. For smoke and cDMARDS, the variables are yes/no for each condition. BMI, Body Mass Index; for this variable grouping was based on normal weight (below 25) versus obese (above 25).

**Table 4:** Bootstrapping analysis for pSTAT3 in CD14+CD16- monocytes.

Bootstrap for Coefficients							
Model		B	Bootstrap (based on 1000 bootstrap samples)				
			Bias	Std. Error	Sig. (2-tailed)	95% Confidence Interval	
						Lower	Upper
1	(Constant)	5.315	-0.082	1.077	0.002	2.890	7.248
	Group	-1.464	0.054	0.594	0.024	-2.471	-0.060
	Smoke	-0.090	0.024	0.700	0.888	-1.238	1.501
	cDMARDS	-0.313	0.003	0.612	0.616	-1.617	0.820
	BMI_NW	0.930	0.044	0.560	0.099	-0.115	2.052

cDMARDs, conventional disease-modifying antirheumatic drugs. For smoke and cDMARDS, the variables are yes/no for each condition. BMI, Body Mass Index; for this variable grouping was based on normal weight (below 25) versus obese (above 25).

**Figure 3:**
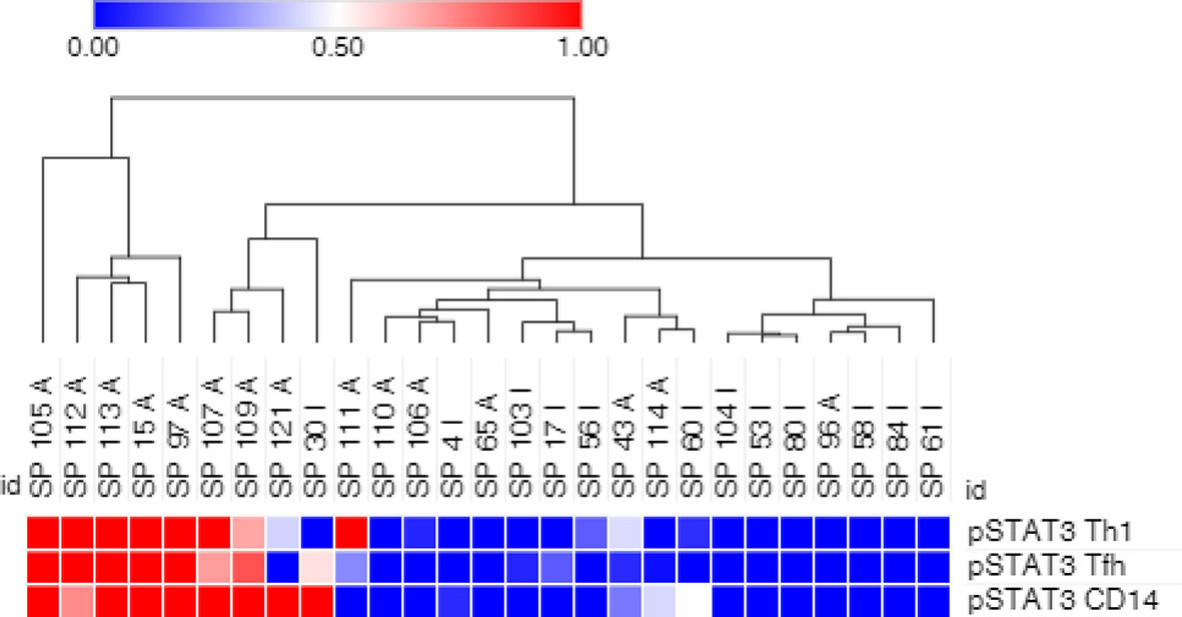
pSTAT3 levels in Th1 and Tfh CD4+ T cells, and in CD14+ monocytes partially cluster PsA samples. Arcsinh transformed geometric mean of pSTAT3 phosphorylated target in Th1 and Tfh CD4+ T cells and CD14+ monocytes was used. Clustering by hierarchical clustering using Euclidean distance with average linkage. A, active PsA; I, inactive PsA.

PsA samples were clustered using the level of pSTAT3 in Th1 and Tfh CD4+ T cells and CD14+ monocytes. The majority of the active samples clusters in 2 adjacent clusters, while half of the inactive samples cluster away from the active samples. Between these 2 groups, there is a mixed group of active and inactive samples (**Figure 3**).

We next examined whether the levels of pSTAT3 in Th1 or Tfh cells CD4+ T cells or in CD14+CD16- monocytes were correlated with tender joints (TJ), swollen joints (SJ), enthesitis or C reactive protein (CRP). There was no correlation between enthesitis or CRP and levels of pSTAT3 in Th1, Tfh or CD14 monocytes (**Supplementary Tables 7**, **8**). Modest negative correlations (r between -0.43 and -0.47) were observed between tender joints or swollen joints and levels of pSTAT3 in Th1 and Tfh cells, although without reaching significance (**Supplementary Figures 3A, B**), while weak negative (r ~ -0.2), non-significant correlations with pSTAT3 in CD14+ monocytes and tender joints or swollen joints were observed (**Supplementary Figure 3C**).

### Comparisons Between Active Psoriatic Arthritis Patients and Active Rheumatoid Arthritis

We also performed a comparison between active PsA and active RA. No significant difference in cell frequency for 16 immune subpopulations was observed between these patient groups (**Supplementary Figure 4**). Levels of phosphorylated proteins were also similar between the two groups (**Supplementary Figure 5**), except for the higher level of pSrc in granulocytes in active PsA (**Supplementary Figure 6**), which was not significant after performing SAM analysis. No correlations were observed between CRP (N=9), TJ or SJ (N=13 for both) in active RA and any phosphoproteins using Spearman’s correlation.

## Discussion

In this study, we describe findings of elevated levels of phosphorylated STAT3 (pSTAT3) in CD4+ T cell subsets and CD14+CD16- (classical) monocytes in active PsA patients, in comparison to patients with inactive disease (inactive PsA). To the best of our knowledge, this is the first report of higher level of pSTAT3 in circulating CD4+ T cells subsets and CD14+CD16- monocytes, measured directly in *ex vivo* cells from PsA patients with active disease. Levels of other signaling proteins analyzed - pSTAT1 and pSrc - were similar between active and inactive PsA patients in all cell types assessed. Elevated pSTAT3 in PsA has been described previously in other tissues or in total T cells. Fiocco et al. (27, 28), found higher levels of pSTAT3, but also of other signaling proteins, on total T cell lysates from synovial fluid of PsA patients compared to T cells from peripheral blood mononuclear cells (PBMC) from healthy donors. These synovial fluid T cells were isolated through a multistep process, which may have affected the signaling profile, and the comparison to circulating cells may be another confounder. In a brief report, Raychaudhuri et al. (29) showed that sorted and activated CD3+ T cells from peripheral blood, upon activation with IL-23, showed increased pSTAT3, but the effect was similar in cells from both PsA patients and healthy controls. Elevated pSTAT3 and pSTAT1 was observed in PsA synovial tissue from PsA in comparison to tissue from osteoarthritis patients (30), indicating that there is activation of the JAK-STAT pathway at inflammatory sites.

In our study, elevated levels of pSTAT3 in active PsA patients, compared to patients with inactive disease, were found in all circulating CD4+ T cells analyzed. These findings agree with results from a murine model showing that expression of STAT3C in CD4+ T cells led to appearance of PsA-like symptoms (14). However, in our study, levels of pSTAT3 in circulating Th17, Treg and Th2 CD4+ T cells were not different in active PsA patients after correction for BMI, smoking (both higher in active patients) and use of c-DMARDs (lower in active PsA). These factors have been shown to affect activation of STAT3 in different directions and may affect subsets of circulating CD4+ cells in distinct ways. Smoke has been shown to activate STAT3 in the lung (31) and cigar smoke extract has been shown to activate STAT3 phosphorylation in a murine macrophage cell line (32). In contrast, c-DMARDS, such as methrotrexate (MTX), have been shown to reduce levels of pSTAT3 (33).

Higher BMI in the active PsA group is consistent with several lines of evidence that indicate that obesity plays an important role in PsA. STAT3 is the main signaling factor of IL-6, and obesity has been shown to chronically activate intracellular JAK-STAT3 signaling through increased levels of IL-6 and leptin (34). Of particular importance to PsA, obesity increases the chance of developing the disease (8, 35). In addition, juvenile PsA patients have been found to have higher rate of obesity compared to polyarticular juvenile idiopathic arthritis (JIA) patients or to the US pediatric population (36). Furthermore, obesity is associated with reduced treatment response in PsA (37). Obesity has been shown to be associated with induction of Th17 cells, but not with Th1 or Treg cells (38, 39). Thus, elevated pSTAT3 in the circulating CD4+ Th17 (and perhaps other helper subsets) in active PsA patients in our study could be partially driven by factors associated with obesity.

For circulating Th1 and Tfh CD4+ T cells, higher expression of pSTAT3 in active PsA compared to inactive PsA was independent of group variables, suggesting an intrinsic role for these cells in PsA. However, in contrast to Th17 cells, Th1 differentiation appears largely independent of STAT3. Indeed, higher expression of IFNγ has been described in STAT3-deficient CD4+ T cells (40), suggesting that STAT3 may inhibit Th1 development. Knocking out STAT3 and mTOR decreased IL-10 production in Th1 cells, but did not affect IFNγ production (41). Although STAT3 does not seem to play a role in Th1 development, increased levels of pSTAT3 in Th1 cells have been found to contribute to organ damage in a model of acute liver injury, suggesting a potential role of these cells in promoting disease pathogenesis (42).

The presence of Th1 cells in psoriatic lesions has been noted for quite some time. However, the role of Th1 cells and IFNγ in psoriasis or PsA is still unclear. In psoriatic plaques, IFNγ producing Th1 cells are abundant, following an initial phase where Th17 predominates (43); expression of IFNγ and IFN-inducible genes is also upregulated. However, a small trial with an anti-IFNγ antibody showed only minimal efficacy in the treatment of psoriasis, in contrast to inhibition of IL-17 activity (44). More recently, Diani et al. described that Th1 (and Tc1) cells expressing the chemokine receptor CXCR3 are enriched in synovial fluid (SF), in contrast to the blood, of PsA patients (45), paralleling a marked increase in CXCL10, the ligand of CXCR3, in the SF of PsA patients. Similar observations were also described in the skin of psoriasis patients (46). These findings suggest a potential role for Th1 (and Tc1) cells that are recruited from the blood in response to CXCL10 chemokine, both in psoriasis and in PsA (47). Expansion of synovial CXCR3+ CD8+ T cells has been also described by Penkava et al. using single-cell sequencing (48). Interestingly, we found that levels of pSTAT3 in circulating Th1 and Tfh cells were negatively correlated with the number of tender or swollen joints, although without reaching statistical significance. Th1 (and perhaps Tfh) cells expressing high levels of pSTAT3 might be recruited to sites of inflammation and contribute to tissue damage in PsA, although how these cells would induce damage, and the exact role of IFNγ in these processes, is currently unclear.

Tfh are a subset of CD4+ T cells in the germinal center that express CXCR5 and provide help to B cells for antibody production; circulating Tfh (cTfh) are considered a counterpart of Tfh, and alterations in the frequency of cTfh have been described in several autoimmune conditions (49). cTfh have been found to be increased in the circulation of patients with psoriasis (49), although pediatric PsA patients had a similar percentage of cTfh in comparison to healthy controls (50). Different subsets of T follicular (Tf) cells have been described, both in secondary lymphoid organs as well as in the circulation (51), including regulatory Tf (Tfr). We did not assess these subsets in our study, and further examination of Tfh associated markers is needed to validate our results (52). Nevertheless, an increase in IL-6/pSTAT3 signaling was found to favor development of Tfh over Tfr (53), suggesting that cTfh with high pSTAT3 might represent preferentially an effector rather than a regulatory subset and thus potentially contribute to disease immunopathology.

We also find that CD14+CD16- monocytes express higher pSTAT3 in active PsA patients. Although the percentage of CD14+ monocytes was not increased in the active compared to the inactive group, enhanced pSTAT3 expression could be related to osteoclast development in active PsA patients (54).

PsA has some similarities with RA, which is also a chronic inflammatory arthritic disease (55), and we did not find differences between active PsA and active RA in the parameters we tested, including pSTAT3 levels, after correction for multiple testing. Constitutive pSTAT3 has been described in CD4+ and CD8+ T cells, as well as in CD14^+^ monocytes from RA patients (56). Jak inhibitors have shown efficacy for treating RA and PsA (57–61), consistent with a potentially important role of STAT3 in both diseases.

STAT3 is able to interact with several different transcription factors in different cell types, and activate diverse sets of genes, leading to distinct phenotypes in different conditions (62). Consistent with this, STAT3 is involved in various cellular processes including inflammation, proliferation, cell growth and differentiation and apoptosis. STAT3 is activated by several pro-inflammatory cytokines, such as IL-6, that may play a role in promoting STAT3 activation in PsA. In preliminary experiments, we found that serum from active PsA patients induces increased phosphorylation of STAT3 in CD4+ T cells from a healthy donor compared to serum from an inactive PsA patient (**Supplementary Figure 7**), suggesting the presence of circulating factors that promote STAT3 activation in active PsA patients.

This study has several limitations. We analyzed a small, heterogeneous sample, and further studies with larger number of patients are needed to confirm our findings. The absence of a healthy control precludes evaluating if PsA inactive patients have returned to healthy levels or are still in a state of compensated inflammation, as we described previously in systemic juvenile idiopathic arthritis (sJIA) (63). Furthermore, diversity within the major CD4+ T cell subsets, as well as within monocytes, was not analyzed and may reveal additional relationships between these cells and PsA. For example, subsets of Th17, such as Th1-like Th17 cells, have been described in autoimmune diseases such as RA (64, 65). These ex-Th17 cells lose expression of IL-17 and produce IFNγ; interestingly, they gain the expression of CXCR3 (66) and could respond to the CXCL10 chemokine. Further studies on the functional capability of these cells will also be necessary to investigate the relevance of the higher pSTAT3 expression in circulating cells in PsA.

In summary, our results show that increased STAT3 signaling in circulating CD4+ T cells, especially Th1 and Tfh, as well as CD14+CD16-monocytes, is associated with active PsA. Evidence suggests that the elevated expression of STAT3 is associated with an effector rather than regulatory function for these cells. These cells may be recruited from the circulation into sites of inflammation and make an important contribution to PsA immunopathology.

## Data Availability Statement

The raw data supporting the conclusions of this article will be made available by the authors, without undue reservation.

## Ethics Statement

The studies involving human participants were reviewed and approved by the IRB at Carmel Medical Center (CMC 0044-11). The patients/participants provided their written informed consent to participate in this study.

## Author Contributions

CM, DZ, and EM designed the research, interpreted the results and wrote the manuscript. CM and SR performed experiments and analyzed data. IL performed statistical analysis. AH and ME performed patient sample collection and processing. DS performed experiments. All authors contributed to the article and approved the submitted version.

## Funding

This work was supported by Pfizer, Israel, Pfizer tracking number W1201524 (DZ, EDM) and the UCSF-Stanford Arthritis Center of Excellence funded by the Great Western Region of the Arthritis Foundation (EDM). The funder was not involved in the study design, collection, analysis, interpretation of data, the writing of this article or the decision to submit it for publication.

## Conflict of Interest

EM reports grants from Novartis outside the submitted work. DZ reports grants from Pfizer during the conduct of the study, personal fees from Ely Lilly, Novartis, and Abbvie, outside the submitted work.

The remaining authors declare that the research was conducted in the absence of any commercial or financial relationships that could be construed as a potential conflict of interest.

The handling editor declared a shared affiliation with DZ at time of review.

## Publisher’s Note

All claims expressed in this article are solely those of the authors and do not necessarily represent those of their affiliated organizations, or those of the publisher, the editors and the reviewers. Any product that may be evaluated in this article, or claim that may be made by its manufacturer, is not guaranteed or endorsed by the publisher.
