# Supplementary material for: High Dimensional Analyses of Circulating Immune Cells in Psoriatic Arthritis Detects Elevated Phosphorylated STAT3
Source: Front Immunol. 2022 Jan 11;12:758418. doi: 10.3389/fimmu.2021.758418 (PMC8787828; doi:10.3389/fimmu.2021.758418)
Supplement: Supplementary file 1 [file DataSheet_1.docx]

**Supplementary Table 1. Antibody panel for CyTOF**

| **Number** | **Label** | **Target** | **Target location** | **Clone** | **Source** |
| --- | --- | --- | --- | --- | --- |
| 1 | 89Y | CD45 | Surface | HI30 | Fluidigm |
| 2 | 141Pr | CD196 (CCR6) | Surface | 11A9 | Fluidigm |
| 3 | 142Nd | CD19 | Surface | HIB19 | Fluidigm |
| 4 | 144Nd | CD11b (Mac-1) | Surface | ICRF44 | Fluidigm |
| 5 | 145Nd | CD4 | Surface | RPA-T4 | Fluidigm |
| 6 | 146Nd | CD8a | Surface | RPA-T8 | Fluidigm |
| 7 | 147Sm | CD11c | Surface | Bu15 | Fluidigm |
| 8 | 149Sm | CD194/CCR4 | Surface | L291H4 | Fluidigm |
| 9 | 150Nd | CD192 (CCR2) | Surface | K036C2 | BioLegend; In house labelling^1^ |
| 10 | 151Eu | CD123 (IL-3R) | Surface | 6H6 | Fluidigm |
| 11 | 152Sm | CD66a/CEACAM1 | Surface | CD66a-B1.1 | Fluidigm |
| 12 | 153Eu | CD7 | Surface | CD7-6B7 | Fluidigm |
| 13 | 155Gd | CD45RA | Surface | HI100 | Fluidigm |
| 14 | 156Gd | CD183 (CXCR3) | Surface | G025H7 | Fluidigm |
| 15 | 158Gd | pStat3 [Tyr705] | Intracellular (phospho) | 4/P-Stat3 | Fluidigm |
| 16 | 159Tb | CD161 | Surface | HP-3G10 | Fluidigm |
| 17 | 160Gd | Tbet | Intracellular | 4B10 | Fluidigm |
| 18 | 161Dy | pSTAT1(Ser727) | Intracellular (phospho) | A15158B | BioLegend; In house labelling^1^ |
| 19 | 162Dy | FoxP3 | Intracellular | PCH101 | Fluidigm |
| 20 | 163Dy | CD235ab (Glycophorin) | Surface | HIR2 | Fluidigm |
| 21 | 164Dy | CD185 (CXCR5) | Surface | RF8B2 | Fluidigm |
| 22 | 165Ho | CD61 | Surface | VI-PL2 | Fluidigm |
| 23 | 166Er | pSrc (Tyr418) ^2^ | Intracellular (phospho) | EP503Y | Abcam; In house labelling^1^ |
| 24 | 167Er | CD27 | Surface | O323 | Fluidigm |
| 25 | 168Er | CD127 (IL-7Ra) | Surface | A019D5 | Fluidigm |
| 26 | 169Tm | CD25 (IL-2R) | Surface | 2A3 | Fluidigm |
| 27 | 170Er | CD3 | Surface | UCHT1 | Fluidigm |
| 28 | 171Yb | CD20 | Surface | 2H7 | Fluidigm |
| 29 | 172Yb | CD38 | Surface | HIT2 | Fluidigm |
| 30 | 174Yb | HLA-DR | Surface | L243 | Fluidigm |
| 31 | 175Lu | CD14 | Surface | M5E2 | Fluidigm |
| 32 | 176Yb | CD56 (NCAM) | Surface | NCAM16.2 | Fluidigm |
| 33 | 209Bi | CD16 | Surface | 3G8 | Fluidigm |

^1^: labeled using Maxpar® X8 Antibody Labeling Kit.

^2^: this antibody may also recognize other members of the SRC family: Lyn (Tyr396); Hck (Tyr410), LcK (Tyr393), Fyn(Tyr419) and Yes(Tyr425).

**Supplementary Table 2. pSTAT3 levels - mean and standard deviation (SD) in CD4+ T cell subsets and monocytes**

| **Subpopulation** | **Mean + SD active PsA** | **Mean + SD inactive PsA** | **Difference between the mean + SEM** |
| --- | --- | --- | --- |
| CD4+ Th1 | 0.4479 + 0.2178 | 0.2080 + 0.0712 | 0.2399 ± 0.0599 |
| CD4+ Th2 | 0.3412 + 0.1613 | 0.2053 + 0.0634 | 0.1359 + 0.0455 |
| CD4+ Th17 | 0.3107 + 0.1234 | 0.2053 + 0.0632 | 0.1054 + 0.0367 |
| CD4+ Tfh | 0.4204 + 0.1971 | 0.2526 + 0.0781 | 0.1678 ± 0.0557 |
| CD4+ Treg | 0.2875 + 0.1111 | 0.1893 + 0.0568 | 0.0982 ± 0.033 |
| CD14+CD16- monocytes | 0.7854 + 0.2590 | 0.4961 + 0.1842 | 0.2893 ± 0.0854 |
| CD14+CD16+ monocytes | 0.5259 + 0.3131 | 0.3177 + 0.1520 | 0.2082 ± 0.092 |

| **Subpopulation** | **95% Confidence Interval** | **R squared** | **P value unpaired t test with Welch’s correction** |
| --- | --- | --- | --- |
| **CD4+ Th1** | 0.1139 to 0.3660 | 0.4771 | **0.0009** |
| **CD4+ Th2** | 0.0407 to 0.2311 | 0.3192 | **0.0076** |
| **CD4+ Th17** | 0.0292 to 0.1816 | 0.2751 | **0.0089** |
| **CD4+ Tfh** | 0.0514 to 0.2843 | 0.3225 | **0.0071** |
| **CD4+ Treg** | 0.02966 to 0.1668 | 0.2895 | **0.0071** |
| **CD14+CD16-monocytes** | 0.1132 to 0.4654 | 0.3169 | **0.0024** |
| **CD14+CD16+ monocytes** | 0.01700 to 0.3994 | 0.1952 | **0.0342** |

**Supplementary Table 3. Level of pSTAT3 - comparisons between PsA active and inactive groups**

**Supplementary Table 4. Bootstrapping analysis for pSTAT3 in Th17 cells**

| **Bootstrap for Coefficients** | | | | | | | |
| --- | --- | --- | --- | --- | --- | --- | --- |
| Model | | B | Bootstrap (based on 999 bootstrap samples) | | | | |
|  |  |  | Bias | Standard Error | Significance (2-tailed) | 95% Confidence Interval | |
|  |  |  |  |  |  | Lower | Upper |
| 1 | (Constant) | 2.063 | .019 | .496 | .003 | 1.118 | 3.141 |
|  | Group | -0.467 | -.002 | .260 | .075 | -1.000 | .059 |
|  | Smoke | -0.137 | -.011 | .257 | .570 | -.653 | .412 |
|  | cDMARDS | -0.243 | -.010 | .225 | .258 | -.744 | .153 |
|  | BMI | 0.183 | .003 | .196 | .340 | -.173 | .575 |

cDMARDs: conventional disease-modifying antirheumatic drugs. For smoke and cDMARDS, the variables are yes/no for each condition. BMI: Body Mass Index; for this variable grouping was based on normal weight (below 25) versus obese (above 25).

**Supplementary Table 5. Bootstrapping analysis for pSTAT3 in Treg cells**

| **Bootstrap for Coefficients** | | | | | | | |
| --- | --- | --- | --- | --- | --- | --- | --- |
| Model | | B | Bootstrap (based on 1000 bootstrap samples) | | | | |
|  |  |  | Bias | Standard Error | Significance (2-tailed) | 95% Confidence Interval | |
|  |  |  |  |  |  | Lower | Upper |
| 1 | (Constant) | 1.686 | 0.014 | 0.354 | 0.004 | 1.056 | 2.487 |
|  | Group | -0.392 | -0.007 | 0.185 | 0.066 | -0.806 | -0.046 |
|  | Smoke | -0.052 | -0.021 | 0.196 | 0.793 | -0.448 | 0.326 |
|  | cDMARDS | -0.122 | 0.011 | 0.187 | 0.527 | -0.502 | 0.221 |
|  | BMI | 0.282 | 0.001 | 0.166 | 0.115 | -0.035 | 0.622 |

cDMARDs: conventional disease-modifying antirheumatic drugs. For smoke and cDMARDS, the variables are yes/no for each condition. BMI: Body Mass Index; for this variable grouping was based on normal weight (below 25) versus obese (above 25).

**Supplementary Table 6. Bootstrapping analysis for pSTAT3 in Th2 cells**

| **Bootstrap for Coefficients** | | | | | | | |
| --- | --- | --- | --- | --- | --- | --- | --- |
| Model | | B | Bootstrap (based on 999 bootstrap samples) | | | | |
|  |  |  | Bias | StandardError | Significance (2-tailed) | 95% Confidence Interval | |
|  |  |  |  |  |  | Lower | Upper |
| 1 | (Constant) | 2.107 | .008^b^ | .457^b^ | .005^b^ | 1.169^b^ | 3.016^b^ |
|  | Group | -0.498 | .004^b^ | .259^b^ | .082^b^ | -1.031^b^ | .034^b^ |
|  | Smoke | -0.018 | -.002^b^ | .298^b^ | .953^b^ | -.570^b^ | .602^b^ |
|  | cDMARDS | -0.355 | -.005^b^ | .271^b^ | .217^b^ | -.954^b^ | .103^b^ |
|  | BMI | 0.330 | .002^b^ | .208^b^ | .136^b^ | -.029^b^ | .734^b^ |

cDMARDs: conventional disease-modifying antirheumatic drugs. For smoke and cDMARDS, the variables are yes/no for each condition. BMI: Body Mass Index; for this variable grouping was based on normal weight (below 25) versus obese (above 25).

**Supplementary Table 7. Spearman’s correlation analysis for enthesitis in active PsA patients and pSTAT3 level**

|  | Enthesitis vs. pSTAT3 Th1 | Enthesitis vs. pSTAT3 Tfh | Enthesitis vs. pSTAT3 CD14 |
| --- | --- | --- | --- |
| Spearman r |  |  |  |
| r | -0.06218 | -0.2544 | -0.09798 |
| 95% confidence interval | -0.5681 to 0.4779 | -0.6872 to 0.3117 | -0.5920 to 0.4496 |
|  |  |  |  |
| p value |  |  |  |
| p (two-tailed) | 0.8256 | 0.3566 | 0.7262 |
| p value summary | ns | ns | ns |
| Exact or approximate p value? | Exact | Exact | Exact |
| Significant (alpha = 0.05) | No | No | No |
|  |  |  |  |
| Number of XY Pairs | 15 | 15 | 15 |

**Supplementary Table 8. Spearman’s correlation analysis for C reactive protein (CRP) in active PsA patients and pSTAT3 level**

|  | CRP vs. pSTAT3 Th1 | CRP vs. pSTAT3 Tfh | CRP vs. pSTAT3 CD14 |
| --- | --- | --- | --- |
| Spearman r |  |  |  |
| r | -0.1276 | 0.08581 | -0.1342 |
| 95% confidence interval | -0.6272 to 0.4463 | -0.4796 to 0.6008 | -0.6312 to 0.4409 |
|  |  |  |  |
| p value |  |  |  |
| p (one-tailed) | 0.3311 | 0.3849 | 0.3226 |
| p value summary | ns | ns | ns |
| Exact or approximate p value? | Exact | Exact | Exact |
| Significant (alpha = 0.05) | No | No | No |
|  |  |  |  |
| Number of XY Pairs | 14 | 14 | 14 |

Supplementary Figure 1. **Gating strategy of immune cell subpopulations in a representative sample**. Two-dimensional plots are shown. Manual gating was performed using FlowJo version 10.5.2. The 16 subpopulations included in the analysis are: Granulocytes, B cells and CD8+ T cells. CD4+ T cells: Tfh, Th17, Th1, Th2 and Treg. pDCs, mDCs, monocytes (all 3 subsets) and NK cells (all 3 subsets).


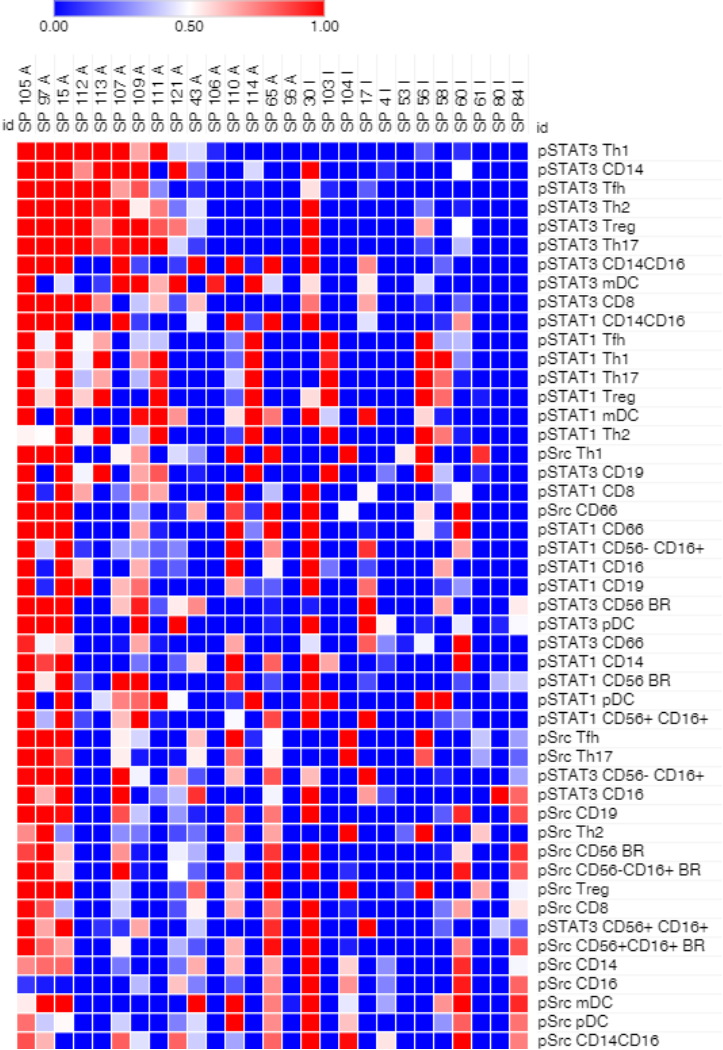


Supplementary Figure 2. **Heat map showing phosphorylation signal in each immune cell subset.** Black bar denotes distinct expression in active compared to inactive group, by two class unpaired SAM analysis, FDR <1% (q<0.01). A: active PsA; I: inactive: PsA.


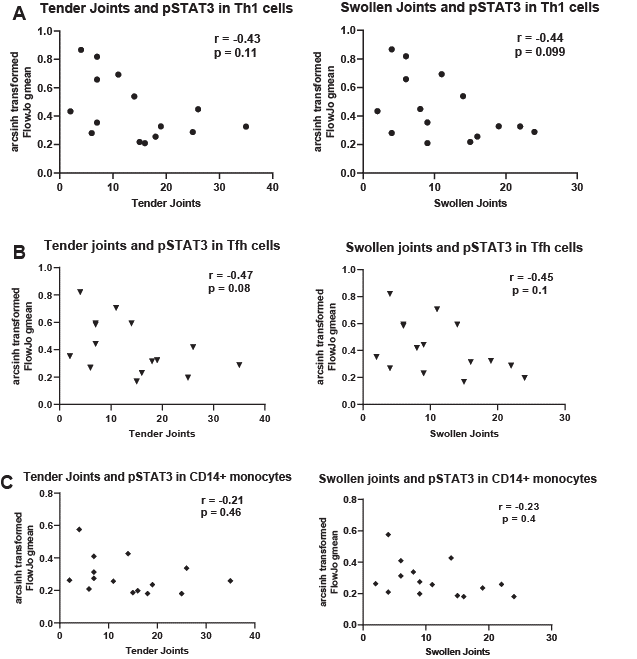


Supplementary Figure 3. **Negative correlations are observed between tender or swollen joints and levels of pSTAT3 in Th1 and Tfh CD4+ T cells in active PsA patients**. A. Spearman correlations between tender or swollen joints and pSTAT3 in Th1 CD4+ T cells. B. Spearman correlations between tender or swollen joints and pSTAT3 in Tfh CD4+ T cells. C. Spearman correlations between tender or swollen joints and pSTAT3 in CD14+ CD16- monocytes. N=15 for all comparisons.

Supplementary Figure 4: **No differences in immune cell frequencies in whole blood between patients with active PsA or active RA. A.** Frequency of granulocytes is expressed as frequency of total white cells. **B.** Frequencies of 15 immune cell subpopulations expressed as frequency of total mononuclear cells (CD45+CD66a-). Data shown as box plots extending from the 25th to 75th percentiles, and the whiskers from the minimum to the maximum point; middle line represents the median. Samples from 15 active PsA and 14 active RA patients were tested. Group to group comparison using unpaired t test with Welch's correction, p>0.05 in all comparisons. Tfh: T follicular helper; mDC: myeloid Dendritic Cell; pDC, plasmacytoid Dendritic cell; MC: Monocyte.

Supplementary Figure 5: **Levels of pSTAT1, pSTAT3 and pSrc in immune subsets are largely similar between active PsA and active RA. A.** Signal intensity of pSTAT1. **B.** Signal intensity of pSTAT3. **C.** Signal intensity of pSrc. Data expressed as arcsinh transformed geometric mean (gmean). Group to group comparison using unpaired t test with Welch's correction, all values p>0.05, except as noted (also see SFig 6). Tfh: T follicular helper; myeloid Dendritic Cell; pDC, plasmacytoid Dendritic cell; MC: Monocyte.

Supplementary Figure 6: **Level of pSrc is increased in granulocytes from active PsA patients in comparison to active RA patients.** Signal intensity of pSrc in granulocytes, expressed as arcsinh transformed geometric mean (gmean). Group to group comparison using unpaired t test with Welch's correction.


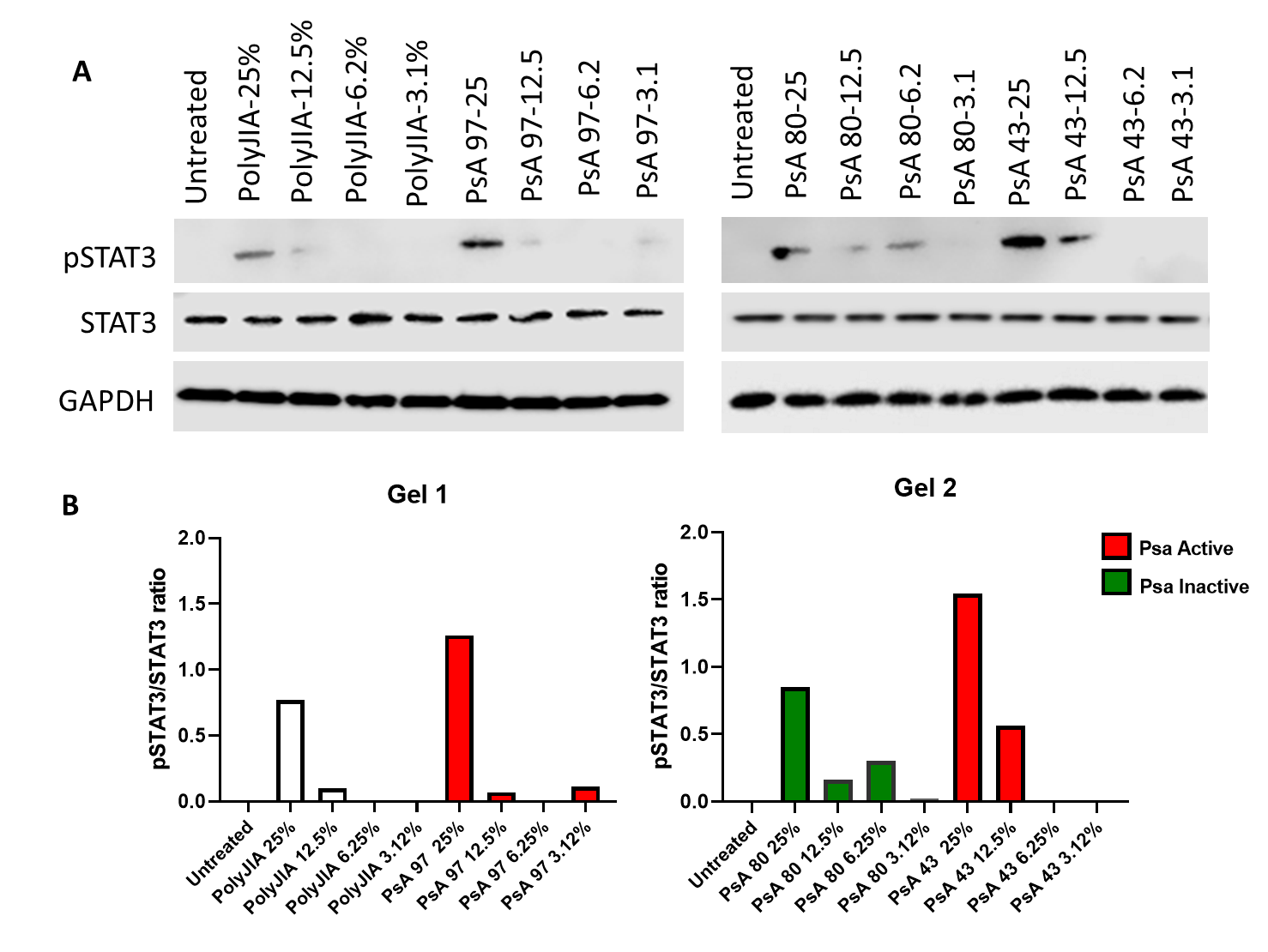


Supplementary Figure 7. **Serum samples from PsA active patients induce activation of STAT3.** A. Western blot for analysis of pSTAT3 is shown for CD4+ T cells from a healthy donor stimulated with serum from PsA patients. Total STAT3 and GAPDH were used as loading control. Isolated CD4+ T cells (1 x 10^6^) from a healthy adult donor were stimulated for 30 min at 37^o^C with decreasing concentrations of serum as indicated (25%-3.1%) in complete RPMI media (with 5% AB human sera) from several PsA patients. PolyJIA: the sample from a patient with polyarticular juvenile idiopathic arthritis, the pediatric counterpart of adult RA was tested as an unrelated disease sample. Cell lysates were prepared and analyzed by Western blot. **B.** Quantification of western blot data in A was analyzed using ImageJ version 1.53e (<http://imagej.nih.gov/ij>).
